# Polarization-independent dielectric gradient near-perfect absorbers for aqueous mid-infrared molecular sensing

**DOI:** 10.1038/s44310-026-00121-9

**Published:** 2026-04-13

**Authors:** Xingye Yang, Tao Jiang, Lina Rohrer, Jonas Biechteler, Andreas Tittl

**Affiliations:** Nano-Institute Munich, Faculty of Physics, Ludwig-Maximilians-Universität München, München, Germany

**Keywords:** Materials science, Optics and photonics, Physics

## Abstract

Mid-infrared molecular sensing offers molecule-specific vibrational fingerprints, yet practical implementation with nanophotonic platforms is challenged by system complexity and strong signal damping in aqueous environments. Dielectric metasurfaces overcome ohmic losses and local heating constraints of metallic resonators and can support high-quality-factor resonances with spectral selectivity, suitable for image-based spectrometer-less sensing. However, their spatially extended near-fields typically render them susceptible to environmental absorption, preventing operation in water. We demonstrate a compact dielectric perfect-absorber metasurface combining C_4_-symmetric quasi-bound states in the continuum (qBICs) with a dual-gradient architecture. The C_4_-symmetric unit cells ensure polarization-independent resonances, enabling efficient utilization of incident light under arbitrary polarization states. The gradient architecture independently controls radiative loss and resonance wavelength, allowing distinct coupling regimes within one metasurface while achieving high absorbance (>0.8). We demonstrate poly(methyl methacrylate) sensing in air with ~20% absorbance envelope modulation under arbitrary polarization. Furthermore, we introduce a sensing configuration utilizing a 700 nm residual thin-water film that preserves qBIC (absorbance ~0.5) near the prominent water absorption peak. This enables the first demonstration of dielectric metasurface-based mid-infrared molecular sensing under a water background, achieving >30% absorbance envelope modulation. This platform extends the utility of dielectric metasurfaces to aqueous environments and supports versatile, spectrometer-less sensing schemes.

## Introduction

Biochemical sensing is essential for environmental monitoring, biology research, and pharmaceutical development^1–3^. Mid-infrared (mid-IR) vibrational spectroscopy provides molecule-specific fingerprint signatures, but the wavelength of these fingerprints is much larger than the molecular dimensions, which weakens light-matter interaction^4^. Nanophotonic cavities can bridge this mismatch by providing strong, localized field enhancement and spectral overlap with molecular resonances, thereby boosting the sensitivity of label-free detection schemes^5^.

For most biologically relevant systems, water is the natural medium: biochemical reactions take place in aqueous environments, cell membranes operate in water, and envisioned point-of-care devices need to work in aqueous background^6^. However, liquid water exhibits a strong absorption band centered at ~1650 cm⁻¹, which damps nearby molecular vibrational features and optical resonances. This makes biochemical sensing based on nanophotonic devices particularly challenging in water, especially in the mid-IR range where many important vibrational fingerprints lie.

To circumvent this central issue for nanophotonic sensing, several strategies have been explored. One approach is to perform sensing in dry conditions^4,7–9^ or to replace H₂O with D₂O^10,11^ whose main absorption band is red-shifted and thus less overlapping with key molecular fingerprints in the mid-IR. However, these methods compromise the natural physiological environment. Dry sensing precludes the study of dynamic processes, while D_2_O exchange can perturb native hydrogen-bonding networks and reaction kinetics, potentially altering the biological system’s functionality^12^. Another route is to use metallic nanoresonators^13–15^, whose near fields are confined to very small, highly localized volumes. In this case, only a limited fraction of the mode interacts with water. Furthermore, illuminating the structures from the substrate side shortens the optical path through the water layer, thereby reducing the detrimental influence of water absorption^11^. Such metallic platforms have enabled the detection of molecules, such as poly(methyl methacrylate) (PMMA) and even real-time amide I and II signals in H_2_O environments^6^.

However, the intrinsic ohmic losses of metals introduce two fundamental limitations. First, they restrict the maximally achievable quality factors (Q, defined as resonance frequency divided by linewidth), leading to broad resonances that are spectrally much wider than the narrow molecular absorption features^16^. In this case, a single broad resonance is typically aligned to a target vibrational line, and the analyte-induced changes in the integrated spectral response are small and thus require spectrometers and careful normalization, which reduces usability and increases system complexity and cost^4^. Second, ohmic dissipation leads to local thermal heating, with temperatures possibly rising to above ~60 °C^17^. Such near-field heating is undesirable for thermally sensitive biomolecules and dynamic reactions that require high powers and long illumination times, limiting the compatibility of metallic nanophotonic platforms with biological systems.

Dielectric nanophotonic sensing platforms can address these limitations. Mie-resonant dielectric resonators are free of ohmic loss, which significantly reduces the intrinsic loss of the photonic cavity and enables high Q factors^18–20^. These high Q modes provide strong near-field enhancement and exhibit narrow resonance linewidths. In contrast to metallic platforms that often rely on a single broad resonance as a probe, dielectric metasurfaces can support a series of narrow resonances that span across the molecular fingerprint region. Because of their high Q factors, these resonances are highly sensitive to small changes in the molecular absorption spectrum: vibrational signatures at different frequencies modulate different resonances with different amplitudes, giving rise to spectral selectivity^4,10^. The large Q factors also mean that analyte-induced changes in the resonances lead to pronounced variations in the overall spectrally integrated intensity. By spatially distributing these detection resonances, one can form a “barcode”, where each pixel encodes the local spectral response through its total intensity. This concept enables image-based spectrometer-less sensing, which greatly improves usability and reduces system complexity^2^. In addition, dielectric cavities avoid the thermal issues associated with ohmic heating, allowing the local temperature to remain essentially constant. Thus, dielectric platforms are preferred when thermal stability of the sensing environment is critical^17^, such as temperature-sensitive DNA-RNA hybridization in SARS-CoV-2 virus detection^21^, single-molecule thermodynamics^22^ and conformational changes of proteins^23^.

However, dielectric resonators tend to exhibit more spatially extended near-field distributions compared to highly localized plasmonic hotspots, rendering them susceptible to environmental loss. When water is introduced into a dielectric metasurface platform, the strong absorption of water can significantly reduce the resonance strength or even completely suppress the resonances, making sensing in water extremely difficult^11,24^. To the best of our knowledge, there has been no demonstration of dielectric metasurface-based sensing in the mid-IR range under an H₂O background^25,26^.

In this work, we realize a compact dielectric perfect-absorber metasurface by combining a C_4_-symmetric unit cell that supports qBICs with a dual-gradient design concept^27^. The C_4_-symmetric unit cells provide polarization-independent resonances, which can efficiently utilize arbitrary polarizations of incident light and relax the illumination requirements^28–30^. Within the qBIC framework, a small in-plane perturbation factor (α) offers a convenient mechanism to tune the radiative loss of the dielectric metasurface and thus the Q factors, while a global scaling factor (S) is used to adjust the resonance wavelength. The dual-gradient concept enables us to encode a large number of resonances within a compact footprint^31^.

Experimentally, we achieve very high absorbance, reaching 0.8 across the wavenumber range 1720 to 1800 cm⁻¹ and confirm polarization-independent behavior via polarization-rotation measurements. We then demonstrate molecular sensing using poly(methyl methacrylate) (PMMA) as a model analyte, resolving its characteristic vibrational fingerprint (around 1730 cm⁻¹) with an absorbance-envelope modulation approaching ~20% in air. Finally, we extend analyte sensing to a H₂O background. Instead of completely immersing the metasurface in water, where the large water volume would strongly absorb and obscure the resonant signal, we allowed the water to recede, measuring the metasurface response under a thin residual water film (estimated to be approximately 700 nm). Under this condition, the qBIC resonances persist due to the significantly reduced optical path length through the thin water film. For a PMMA layer of about 7 nm thickness, we demonstrate, for the first time to our knowledge, sensing with dielectric metasurface under a water absorption background spectrum. By normalizing the absorbance spectral envelope to a reference envelope (without PMMA), we obtain a clear PMMA characteristic resonance with its modulation exceeding 30%. Our work extends the applicability of dielectric metasurfaces and proposes a new approach that can be further explored.

## Results

### Dielectric Polarization-independent gradient near-perfect absorber metasurface design

**Fig. 1:**
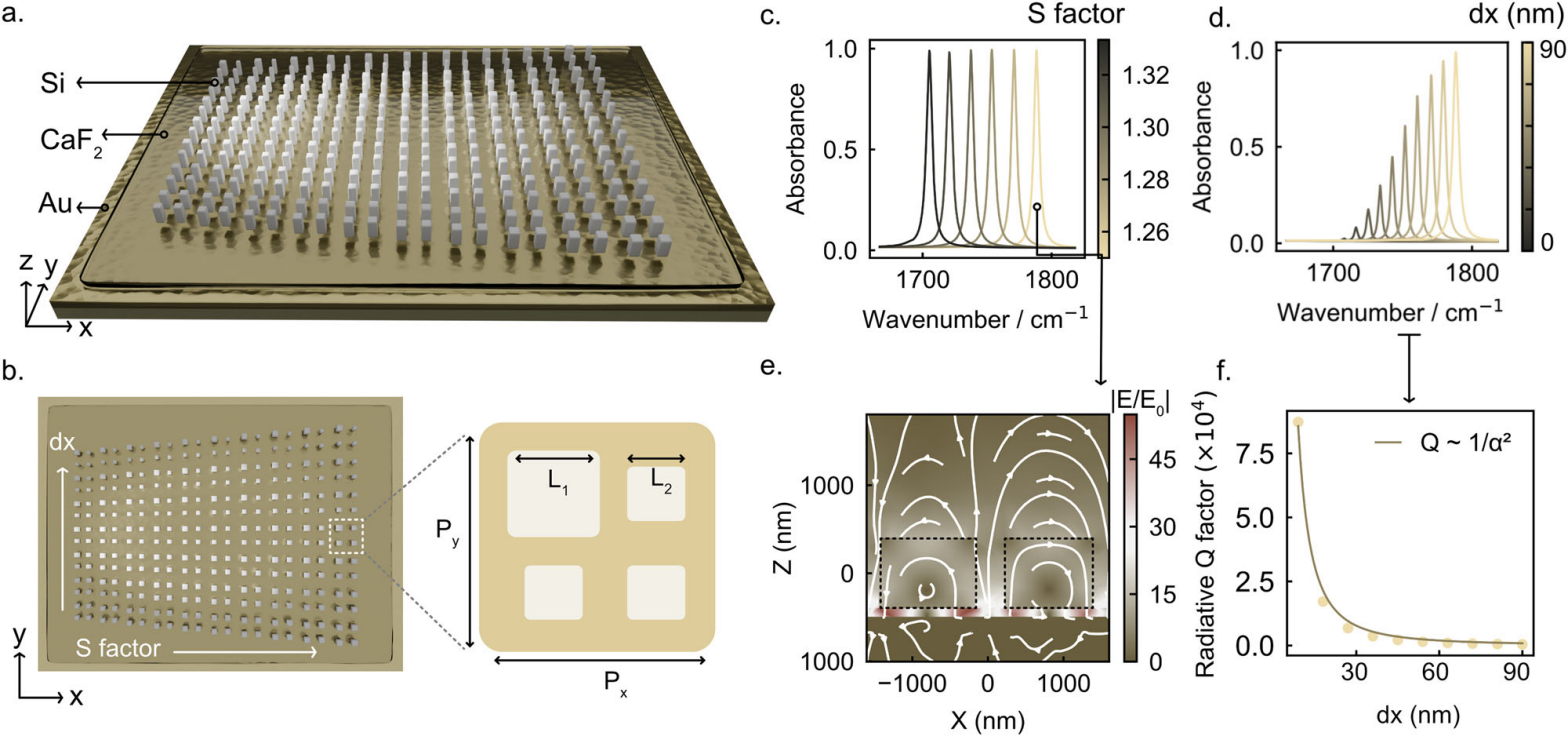
Design of the polarization-independent dual-gradient Si metasurface perfect absorber. **a** Schematic of the multilayer structure consisting of a 200 nm Au back reflector, a 100 nm CaF₂ spacer, and Si nanoresonators (height: 800 nm). **b** Unit cell geometry featuring four dielectric square blocks, with one block defined by L_1_ and the remaining three by L_2_, where dx = L_1_ − L_2_ denotes the geometric perturbation. A scaling factor S is applied to in-plane dimensions to shift the resonance frequency. The dual-gradient arrangement varies S from 1.00 to 1.33 along x axis and dx from 0 to 90 nm along y axis. **c** Simulated absorbance spectra (1 − Reflectance) for the dx = 90 nm, showing narrowband dielectric resonances with very high absorbance and uniform spectral coverage from 1700 to 1800 cm⁻¹. **d** Spectra for S = 1.25, with dx varying from 0 to 90 nm with stepsize 9 nm. **e** Near-field electric-field distribution |E/E_0_| in the X–Z plane for the S = 1.25 in **c**, indicating strong field confinement near the Si resonators and the spacer layer. **f** Radiative quality factor Q_rad_ as a function of dx (α = dx × S), exhibiting an inverse-quadratic dependence characteristic of quasi-BIC resonances.

We first validated the design of the gradient Si metasurface perfect absorber using CST simulations. As shown in Fig. 1a, the device is composed of three layers on a substrate: a 200 nm thick Au back reflector, a 100 nm thick CaF₂ spacer layer, and an array of dielectric nanoresonators made of Si with a height of 800 nm. The unit cell design is illustrated in the right panel of Fig. 1b. The periodicity is P_x_ = P_y_ = 2580 nm, and each unit cell consists of four square blocks. One block has a side length L_1_ = 1020 nm, while the other three blocks have a side length L_2_. The perturbation is introduced through a perturbation parameter α = dx × S = (L_1_ − L_2_) × S. A scaling factor S is applied to all in-plane dimensions (excluding the height), enabling a uniform spectral shift of the resonance wavelength.

This unit cell is implemented in a dual-gradient configuration, as shown in Fig. 1b: dx increases from 0 to 90 nm along the y-axis, while S increases from 1.00 to 1.33 along the x-axis. Despite the weakly perturbed quasi-periodic arrangement, a well-defined resonance is maintained at each unit-cell position^27^, and the variation of geometric parameters results in multiple distinct resonances in a compact footprint. For instance, selecting the row with dx = 90 nm and scanning S from 1.25 to 1.33 yields a series of narrow spectral resonances with a total Q factor of 368 in simulations (Fig. 1c, f) and around 75 in experiments (see supplementary information Figure S4d). Due to the absence of ohmic loss in the dielectric resonators, the linewidth remains narrow and the absorbance (defined as 1 – reflectance) approaches unity. With increasing S, the resonance wavenumber shifts uniformly from 1700 cm⁻¹ to 1800 cm⁻¹, indicating effective coverage of the characteristic vibrational band of the target analytes. The near-field distribution of the S = 1.25 resonance is shown in Fig. 1e, where the electric-field enhancement |E/E_0_| in the X-Z plane reaches values exceeding 45 times near the resonators and spacer layer, suggesting strong light-matter interaction.

Along with the y-axis gradient, the variation of dx can be understood within the framework of bound states in the continuum (BICs). When dx = 0, the structure supports a BIC with negligible radiative loss. Introducing finite dx folds the guided modes into the Γ point via Brillouin zone folding, enabling radiation leakage and forming quasi-BIC resonances. As dx increases, the radiative loss increases, resulting in a lower Q factor and higher mode amplitude, as shown in Fig. 1d. Extracted radiative Q factors follow an inverse-quadratic dependence on α, consistent with the characteristic behavior of qBICs.

Through this dual-gradient design, we achieve a Si perfect absorber that provides both: (i) spectral coverage of the desired molecular absorption band via in-plane scaling along the x-direction, and (ii) tunable radiative coupling along the y-direction, enabling the coexistence of critical-coupling (perfect absorption) and under-coupled regions on the same metasurface. The latter exhibits stronger analyte-induced absorbance modulation, making it particularly suitable for molecular sensing applications^7^. The coexistence of all these regimes on one metasurface significantly simplifies the fabrication process and the subsequent sensing procedure (see experiment results in supplementary information Figure S4 and S5).

### Experimental validation of gradient polarization-independent near-perfect absorber

**Fig. 2:**
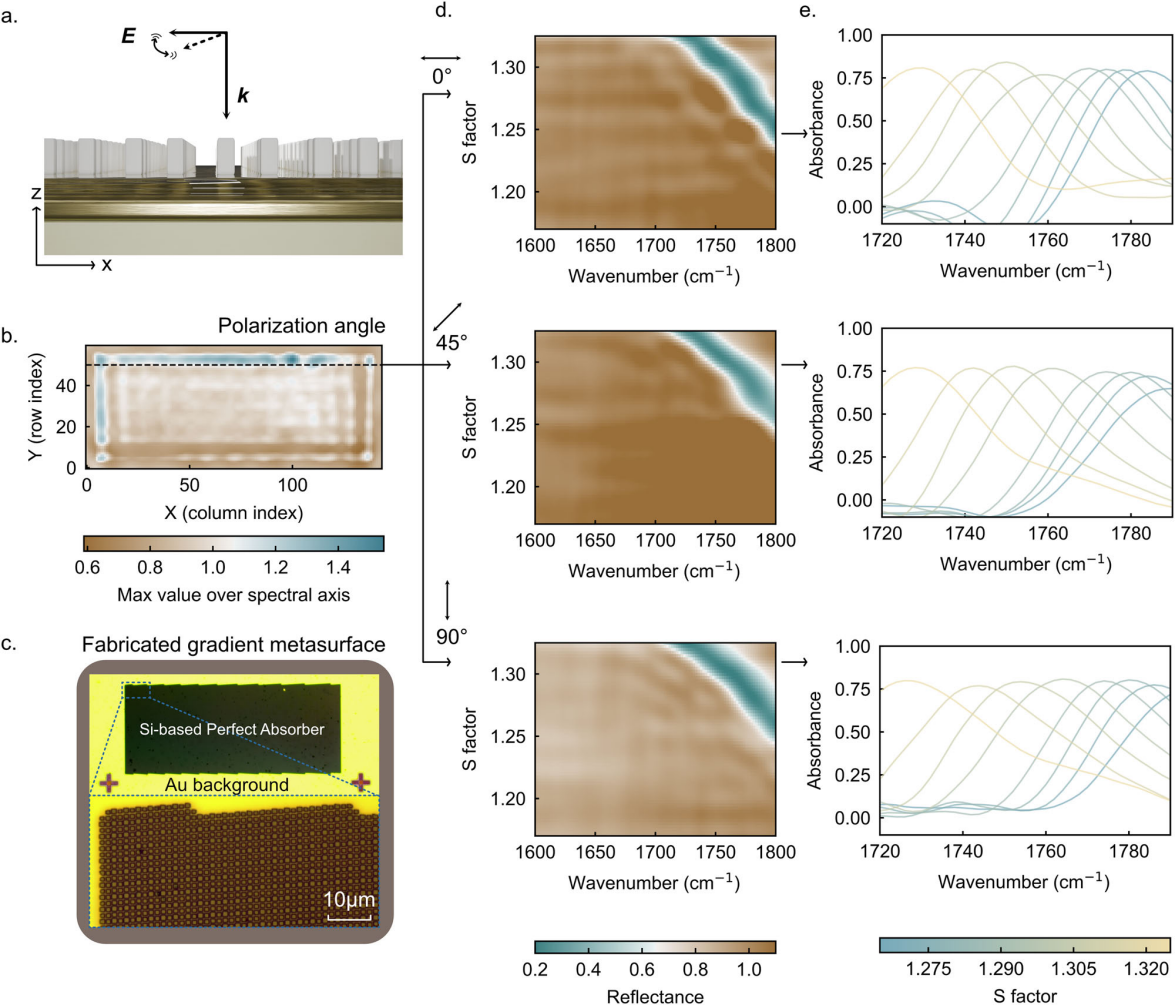
Experimental validation of polarization-independent near-perfect absorption in the dual-gradient Si metasurface. **a** Schematic of mid-infrared measurement with normal incidence illumination. **b** Metasurface in the microscope from an overview. **c** Microscope image of the fabricated metasurface with a zoom in on the edge area where slight unit cell misalignment appears due to in-plane scaling. **d** Two-dimensional reflectance map extracted along the dashed line in **b**, where each pixel corresponds to a different scaling factor S. **e** Experimental absorbance spectra at resonant positions for polarization angles of 0°, 45°, and 90°, demonstrating stable resonances with ~0.8 absorbance within 1720–1800 cm⁻¹, confirming polarization-independent near-perfect absorption. The smoother envelopes under 45° and 90° polarization are attributed to perfect unit cell alignment along the y-direction, enabling better collective mode excitation.

We experimentally fabricated the proposed metasurface using a standard electron-beam lithography (EBL) process (see Methods) and characterized its response with a mid-infrared hyperspectral microscope (Spero). As illustrated in Fig. 2a, normally incident light is projected onto the metasurface along the -z direction. The Spero system records an image at each wavenumber using a tunable quantum cascade laser (QCL) source and reconstructs a hyperspectral datacube containing the full spectral information for each pixel. Figure 2b shows the measured metasurface area, where every pixel provides a complete reflectance spectrum. We extracted spectra along the black dashed line, where each pixel corresponds to a different S factor, and present the results as a two-dimensional (2D) reflectance map in Fig. 2d. The corresponding absorbance spectra of different S factors are plotted in Fig. 2e.

Across the 1720–1800 cm⁻¹ range, all extracted spectra reach an absorbance level of 0.8, confirming the experimental realization of a near-perfect absorber. We note that the spectral envelope exhibits slight fluctuations, which we attribute to the relative positional offsets between adjacent unit cells introduced by the in-plane scaling (S factor) in the dual-gradient design. In the gradient metasurface, when extracting spectra along a line of constant perturbation factor (i.e., along a horizontal line in Fig. 1b and experimentally in Fig. 2b), the scaling factor varies along the x-direction. As a result, unit cells located near the center of the metasurface remain well aligned, whereas those closer to the edges experience increasing relative positional offsets due to cumulative scaling (Fig. 2c). Along such a line, neighboring unit cells are therefore not perfectly aligned, leading to small perturbations of the supported collective resonance modes under x-polarized illumination. These perturbations manifest as fluctuations in the spectral envelope.

Given the C_4_-symmetry of the unit cell, we further measured the device response under different polarization angles, including 0°, 45°, and 90° (upper, middle and bottom panels in Fig. 2d, e). Under all polarization states, the resonances retain stable amplitudes around 0.8, validating the polarization-independent behavior of the absorber and reducing complexity requirements for the illumination optics in practical deployment and increasing the illumination efficiency by utilizing the full energy of the incident light regardless of polarization angle. We also observe that the spectral envelopes under 45° and 90° polarization appear smoother. This can be attributed to the high unit-cell alignment along the y-direction, where no positional mismatch is introduced by the perturbation factor α. As a result, resonators within each column couple more efficiently and form a stronger collective mode. Even at the boundary of the metasurface, the unit cells maintain high absorbance values (~0.8) together with improved envelope uniformity. The preservation of high absorbance near the boundary also indicates a strong robustness of the supported resonance mode. This is attributed to the excitation of a higher-order magnetic quadrupole eigenmode^18,32–35^, which exhibits a more complex field distribution and can lead to stronger inter-cell coupling compared to a simple anti-parallel dipole. Our temporal coupled-mode theory (TCMT) fitting confirms that the spectra are measured in the critical-coupling regime (supplementary information Figure S4). However, due to the finite angular components in the illumination and other practical experimental factors, the peak absorption saturates at ~0.8 (supplementary information Figure S6).

### Polarization-independent sensing

**Fig. 3:**
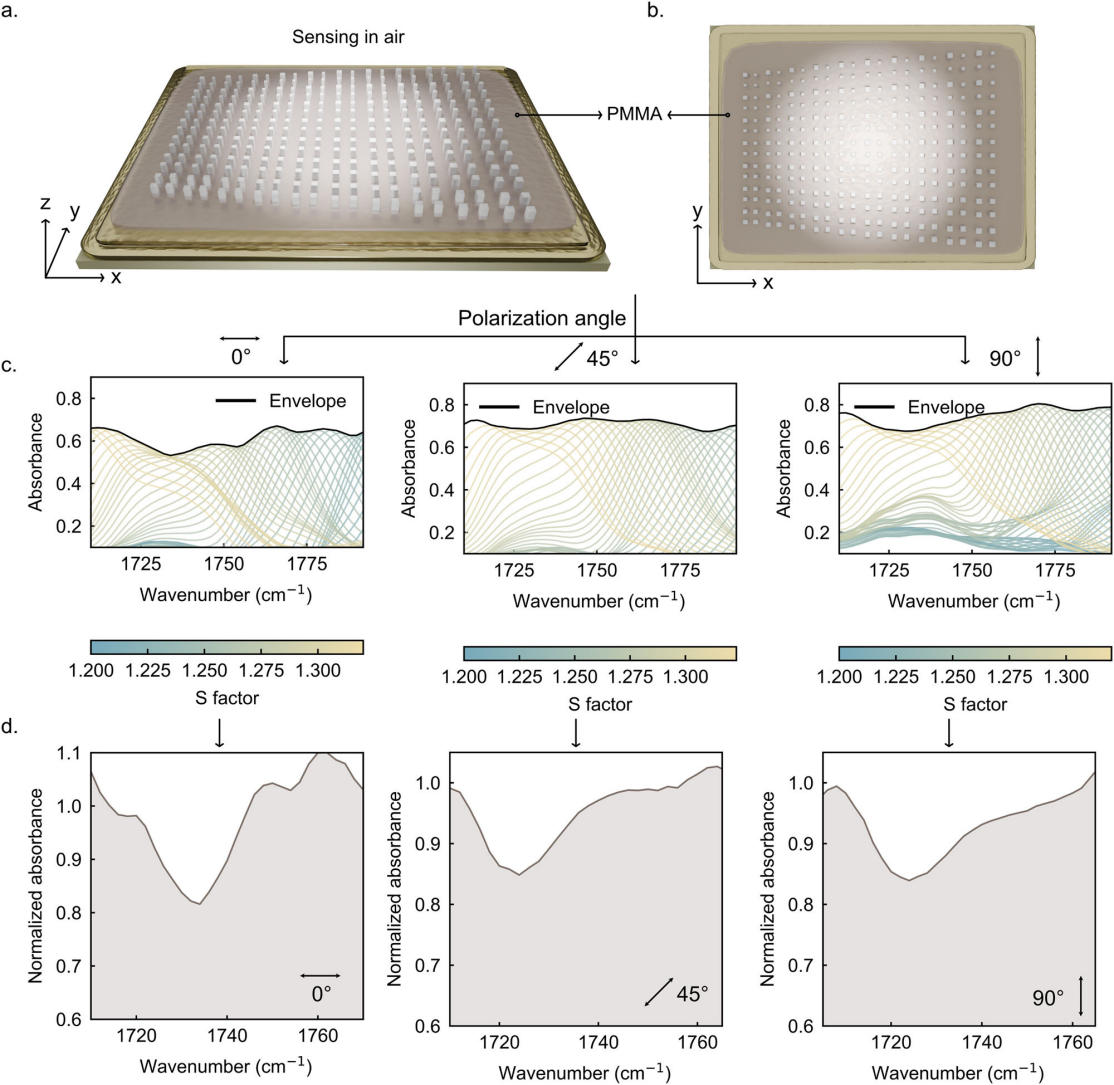
Polarization-independent molecular sensing performance of the dual-gradient Si metasurface absorber using PMMA as the analyte. **a**, **b** Schematic of the measurement configuration with a ~ 4 nm PMMA overlayer deposited on the metasurface via spin coating. **c** Experimental absorbance envelopes extracted along the S-gradient under incident polarization angles of 0°, 45°, and 90°, showing clear perturbations near the PMMA vibrational resonance. **d** Normalized absorbance envelopes obtained by dividing the PMMA-covered envelopes by their respective bare-device references, demonstrating consistent PMMA signatures with ~20% resonance modulation across all polarization states.

To validate the polarization-independent sensing capability of the dielectric perfect absorber, we selected PMMA as an analyte due to its widespread adoption as a benchmark material in optical sensing^36–38^ and the convenient control of film thickness via spin-coating solutions with different concentrations. A thin PMMA layer of approximately 4 nm was deposited on the metasurface, as illustrated in Fig. 3a, b. The structure was then measured in air under three different incident polarization angles (0°, 45°, and 90°), focusing on the resonance region that overlaps with the characteristic absorption band of PMMA. Specifically, we extracted spectra from a pixel line slightly below the one used in Fig. 2b. This selected region lies in the slightly under-coupled regime where molecular sensing is more favorable^7^ while still maintaining sufficiently strong resonance features. The absorbance spectra along the S-gradient were obtained for each polarization state, and their envelopes were plotted in Fig. 3c. Pronounced perturbations in the envelopes appear at the PMMA vibrational absorption peak, indicating analyte-induced resonance modulation.

We then normalized these envelopes by referencing them to their respective polarization-matched bare-device envelopes (measured prior to PMMA deposition). The resulting normalized absorbance envelopes are shown in Fig. 3d. Clear and consistent PMMA signatures with ~20% resonance modulation amplitudes are observed under all polarization angles, demonstrating robust sensing performance. It is noted that for 0°-polarized excitation, the extracted resonance positions and baseline fluctuations show larger deviations than those observed for 45° and 90° excitation. This behavior can be attributed to the geometric scaling inherent to the dual-gradient design, which gradually introduces unit-cell misalignment along the x-direction and therefore reduces the uniformity of inter-cell coupling. In contrast, resonances excited along the y-directed field component are supported by well-aligned unit-cell columns, resulting in more stable resonance positions and amplitudes. Additionally, the PMMA-related resonances retrieved under 45° and 90° excitation exhibit a small red-shift to ~1723 cm⁻¹. This slight variation in the spectral position of the PMMA signature is attributed to small polarization-induced modulations of the near-field amplitudes associated with the qBIC resonances, which modify the coupling with the PMMA vibrational mode and lead to slight changes in lineshape due to Fano interference^39^. Nevertheless, the distinct modulation of the normalized absorbance envelopes across all polarization states confirms the sensing potential of this metasurface in polarization-independent applications.

### Sensing PMMA with the dielectric metasurface under a water background

**Fig. 4:**
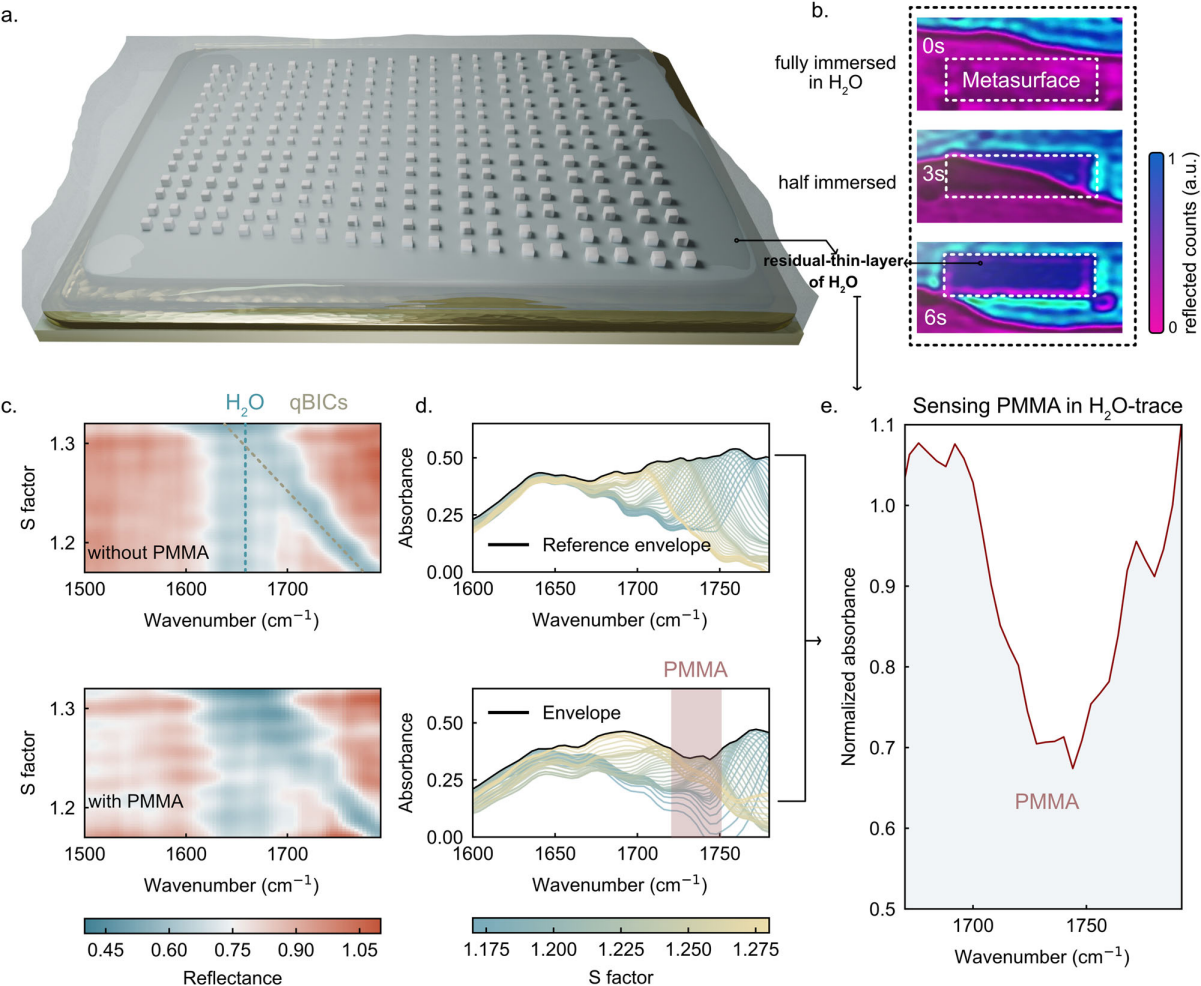
Demonstration of PMMA aqueous sensing enabled by a residual thin water film on the dual-gradient Si metasurface. **a** Conceptual illustration of the sensing scheme: a bulk water layer is applied and subsequently flows over the metasurface, leaving behind a residual thin water film that enables sensing under a water-background condition. **b** Top-view experimental snapshots showing the transition from full water immersion (0 s) to partial exposure (3 s) and final emergence (6 s), while a stable thin water film remains on the metasurface surface. **c** Spectral reflectance colormaps along the S-gradient in the water-film-only reference case (upper panel) and adding PMMA-coating case (~7 nm) (lower panel). **d** Respective corresponding absorbance envelopes showing persistence of qBIC resonances near the water absorption band (~1650 cm⁻¹) with strong amplitude (~0.5) across 1675–1792 cm⁻¹ and spectral selective modulation by PMMA. **e** Normalized absorbance envelope obtained by dividing PMMA modulated envelope with the water-film-only reference envelope, revealing a clear analyte-induced resonance modulation (>30%) around the PMMA characteristic vibrational position, confirming mid-IR molecular sensing under a water background.

We discovered that by immersing the metasurface in water and subsequently allowing the water layer to recede, a thin residual water film remained adsorbed on the surface, enabling molecular sensing under a water background, as illustrated in Fig. 4a. The top-view experimental configuration is shown in Fig. 4b: a droplet of water was dispensed onto the metasurface (a CaF₂ slip was then placed on top to planarize the water layer). At 0 s, the metasurface was fully submerged; the strong water absorption suppressed all resonances. At 3 s, as water evaporated and the waterline receded, the metasurface gradually emerged until it became fully exposed (6 s), while still retaining a thin residual water film, visible through an increased reflected count (bluish purple). This thin film allows vibrational sensing in the presence of water.

We first examined the sensing behavior without PMMA. Figure 4c (upper panel) and 4 d (upper panel) show hyperspectral reflectance colormaps and absorbance envelopes across the S-gradient. Remarkably, the water absorption peak at ~1650 cm⁻¹ (indicated with a blue dashed line) coexists with the qBIC resonances (indicated with a gold dashed line) which persist and shift from 1675 to 1800 cm⁻¹ as S increases. This indicates that qBICs remain functional near the water-absorption band, maintaining strong resonance amplitude. A consistent absorbance amplitude of ~0.5 is maintained across 1675–1792 cm⁻¹, despite slight attenuation near 1650 cm⁻¹. Moreover, the water absorption peak exhibits highly consistent amplitude and spectral position across all S-factor locations, indicating that the residual water film is uniformly distributed over the metasurface. To the best of our knowledge, such coexistence of designed dielectric qBIC resonances with the H₂O absorption band in the mid-IR has not been reported previously. By contrast, earlier studies showed resonance suppression near the water band in bulk water background, preventing PMMA detection under aqueous conditions^11^. Importantly, the residual water film remains relatively stable for ~180 s, providing a practical time window for sensing under a water background (supporting information Figure S1). By performing simulations under different water layer thicknesses and comparing them to the experimental data in Fig. 4d, we can estimate that the thickness of the residual water layer is around 700 nm (supporting information Figure S2).

We then introduced PMMA as the analyte, depositing a ~ 7 nm film followed by 10 min UV-ozone treatment to enhance hydrophilicity. The measurement is conducted under the 700 nm residual water film condition, as established earlier, rather than full immersion, producing the resonance signals shown in Fig. 4c (lower panel) and 4 d (lower panel). This scenario is close to established aqueous SEIRA sensing schemes, where the target molecules are present as surface-associated layers such as biomimetic membranes or lipid assemblies forming on the sensor surface^6^. Clear absorbance envelope perturbations appear at the PMMA absorption band (red-highlighted region) together with clear water absorption band. Upon normalization by the water-only reference envelope, the normalized absorbance shown in Fig. 4e reveals a strong analyte-induced modulation >30% at the PMMA vibrational position. These signatures represent, to our knowledge, the mid-infrared detection of PMMA using a dielectric metasurface under water-background, which has not been achieved in prior work.

Our approach expands the applicability of dielectric metasurfaces by enabling a new approach based on the residual thin water film coverage. It provides a practical aqueous-background configuration in which high-Q dielectric resonances retain spectrally selective molecular sensing capability. However, for applications requiring precise quantitative control of bulk water properties (e.g., pH-dependent measurements or concentration-sensitive aqueous analysis), further environmental stabilization would be necessary. Future integration with precise surface hydrophilicity treatment and microfluidics could further stabilize and control the water-layer thickness. For example, by using a flow of humidified N₂ to remove the bulk water and slow down the evaporation of residual water layer to obtain a more stable and better-defined water layer on the surface. Furthermore, the spatially distributed resonances and spectral selectivity of dual-gradient architecture allow compatibility with image-based, spectrometer-less sensing platforms, with potential combination with AI-driven molecular identification. Finally, the inherent polarization-independent response reduces optical system complexity and enhances practicality for bio-chemical sensing environments.

## Discussion

In this work, we presented a dielectric near-perfect absorber metasurface platform that leverages qBIC resonances in a C_4_-symmetric unit cell combined with a dual-gradient design. The C_4_-symmetry ensures polarization-independent operation and thus simplifies the optical excitation configuration. The qBIC framework enables independent tuning of the radiative loss and Q-factor through a controllable in-plane geometric perturbation and the resonance wavelength is continuously shifted by applying a scaling factor within a compact dual-gradient design. Experimentally, very high absorbance (>0.8) is observed from 1720 to 1800 cm⁻¹. Under varying polarization states (0°-90°), stable absorption features and consistent sensing performance confirm the polarization-independent characteristics of the device, retrieving clear PMMA (4 nm thickness) absorption signatures near 1730 cm⁻¹ with ~20% modulation. Interestingly, we demonstrate mid-infrared molecular sensing under a water-background condition. By utilizing a residual thin water film layer instead of full immersion where conventional dielectric metasurfaces lose spectral information due to strong H₂O absorption, the qBIC resonances remain discernible with an absorbance of ~0.5 across 1675-1792 cm⁻¹. This enables the first experimental detection of PMMA using a dielectric metasurface under H_2_O absorption background, yielding >30% modulation in the normalized absorbance envelopes. Overall, our approach expands the applicability of dielectric metasurfaces for biochemical analysis, introducing a new approach based on residual thin film water backgrounds. The design is potentially compatible with microfluidics for more stabilized water-layer control, and the spectral selectivity of dielectric platform offer pathways toward imaging-based, spectrometer-less molecular detection with potential AI-assisted analysis. The polarization-independent nature of the device further reduces illumination complexity, suggesting its promise for highly integrated molecular sensing technologies.

## Methods

### Numerical simulations

Electromagnetic simulations were carried out using CST Studio Suite (Simulia), a commercial finite-element solver operating in the frequency domain. Periodic boundary conditions were applied and adaptive mesh refinement was enabled to ensure numerical convergence. The CaF₂ substrate was treated as lossless and non-dispersive over the wavelength range of interest, with a constant relative permittivity of ε = 1.9. The real part of the permittivity of amorphous silicon was set to ε = 11.9.

### Sample fabrication

A 200 nm gold layer was first deposited onto a silica substrate, followed by the deposition of a 100 nm CaF₂ layer, both using thermal evaporation (Angstrom Engineering). Subsequently, an 800 nm amorphous silicon film was deposited on the CaF₂ layer via plasma-enhanced chemical vapor deposition (PECVD) using a PlasmaPro 100 system (Oxford Instruments). Nanostructuring was performed by spin-coating a 400 nm layer of positive electron-beam resist (ZEP520A, Zeon Corporation), followed by the application of a conductive polymer layer (ESPACER, Showa Denko K.K.). Electron-beam lithography was conducted using an eLINE Plus system (Raith) at an acceleration voltage of 20 kV with a 20 μm aperture. After exposure, the resist was developed in amyl acetate, followed by a methyl isobutyl ketone and isopropyl alcohol (1:9) rinse. A 60 nm chromium layer was then deposited, and lift-off was performed using Microposit Remover 1165 (Microresist). The remaining chromium pattern served as a hard mask for subsequent reactive ion etching, carried out using SF₆ and argon gases. Finally, the chromium mask was removed using TechniEtch Cr01 (MicroChemicals).

### Molecular sensing

For molecular sensing experiments, the dual-gradient metasurface was coated with PMMA (495k molecular weight, diluted in anisole) via spin coating at 3000 rpm for 1 min, resulting in a uniform polymer layer. The coated samples were subsequently baked at 180 °C for 3 min to fully solidify the PMMA film. Between consecutive measurement cycles, the PMMA layer was removed by sequential immersion in acetone and isopropyl alcohol baths. The thickness of the PMMA films was estimated using a polynomial calibration curve^27^: $d=1.237{c}^{3}+4.773{c}^{2}+10.88c+0.9635,$ where *d* denotes the PMMA thickness in nanometers and *c* represents the PMMA concentration in percent. For sensing measurements performed in air, a PMMA concentration of 0.25% was used, corresponding to a film thickness of approximately 4 nm. For measurements conducted in H₂O, the PMMA concentration was increased to 0.5%, yielding a thickness of approximately 7 nm. The higher concentration was selected to compensate for the reduced hydrophobicity of the PMMA layer following a 10 min UV–ozone treatment, which was applied to improve surface wettability while avoiding complete degradation of the polymer film.

### Optical characterization

Optical measurements were carried out using a Spero mid-infrared spectral imaging microscope (Daylight Solutions), schematically shown in Supplementary Fig. S3. The system was equipped with a 4× objective (numerical aperture 0.15), providing a field of view of approximately 2 × 2 mm^2^ with a detector array of 480 × 480 pixels and an effective pixel size of ~4 × 4 µm^2^. Illumination was provided by three tunable quantum cascade lasers covering the spectral range from 5.6 to 10.5 µm with a spectral resolution of 4 cm⁻¹ in our experiments. The laser output was linearly polarized. Different incident polarization orientations were realized by rotating the sample relative to the fixed polarization axis of the excitation beam.

## Supplementary information

Supplementary information

## Data Availability

The data that support the findings of this study are available from Zenodo [10.5281/zenodo.19348019].

## References

[1] Neubrech, F., Huck, C., Weber, K., Pucci, A. & Giessen, H. Surface-enhanced infrared spectroscopy using resonant nanoantennas. *Chem. Rev.* **117**, 5110–5145 (2017). 28358482 10.1021/acs.chemrev.6b00743

[2] Chung, T., Wang, H. & Cai, H. Dielectric metasurfaces for next-generation optical biosensing: a comparison with plasmonic sensing. *Nanotechnology* **34**, 402001 (2023). 10.1088/1361-6528/ace117 PMC10416613 37352839

[3] Tittl, A., John-Herpin, A., Leitis, A., Arvelo, E. R. & Altug, H. Metasurface-based molecular biosensing aided by artificial intelligence. *Angewandte Chemie International Edition* **58**, 14810–14822 (2019). 31021045 10.1002/anie.201901443

[4] Tittl, A. et al. Imaging-based molecular barcoding with pixelated dielectric metasurfaces. *Science (1979).* **1109**, 1105–1109 (2018). 10.1126/science.aas9768 29880685

[5] Dregely, D., Neubrech, F., Duan, H., Vogelgesang, R. & Giessen, H. Vibrational near-field mapping of planar and buried three-dimensional plasmonic nanostructures. *Nat. Commun.* **4**, 2237 (2013). 23892519 10.1038/ncomms3237 PMC3731659

[6] Rodrigo, D. et al. Resolving molecule-specific information in dynamic lipid membrane processes with multi-resonant infrared metasurfaces. *Nat. Commun.* **9**, 2160 (2018). 29867181 10.1038/s41467-018-04594-x PMC5986821

[7] Wang, J., Weber, T., Aigner, A., Maier, S. A. & Tittl, A. Mirror-coupled plasmonic bound states in the continuum for tunable perfect absorption. *Laser Photon. Rev.* **17**, 2300294 (2023).

[8] Autore, M. et al. Boron nitride nanoresonators for phonon-enhanced molecular vibrational spectroscopy at the strong coupling limit. *Light Sci. Appl.* **7**, 17172 (2018). 30839544 10.1038/lsa.2017.172 PMC6060053

[9] Rodrigo, D. et al. Mid-infrared plasmonic biosensing with graphene. *Science* **349**, 165–168 (2015). 26160941 10.1126/science.aab2051

[10] Barkey, M. et al. Pixelated high-Q metasurfaces for in situ biospectroscopy and artificial intelligence-enabled classification of lipid membrane photoswitching dynamics. *ACS Nano* **18**, 11644–11654 (2024). 38653474 10.1021/acsnano.3c09798 PMC11080459

[11] Jiang, T. et al. A comparative analysis of plasmonic and dielectric metasurface sensing platforms powered by bound states in the continuum. *Adv. Funct. Mater.* **36**, e16021 (2025).

[12] Giubertoni, G., Bonn, M. & Woutersen, S. D2O as an imperfect replacement for H2O: problem or opportunity for protein research?. *J. Phys. Chem. B* **127**, 8086–8094 (2023). 37722111 10.1021/acs.jpcb.3c04385 PMC10544019

[13] Limaj, O. et al. Infrared plasmonic biosensor for real-time and label-free monitoring of lipid membranes. *Nano Lett.* **16**, 1502–1508 (2016). 26761392 10.1021/acs.nanolett.5b05316

[14] John-Herpin, A., Tittl, A. & Altug, H. Quantifying the limits of detection of surface-enhanced infrared spectroscopy with grating order-coupled nanogap antennas. *ACS Photonics* **5**, 4117–4124 (2018). 30828588 10.1021/acsphotonics.8b00847 PMC6390698

[15] Huang, S. H., Li, J., Fan, Z., Delgado, R. & Shvets, G. Monitoring the effects of chemical stimuli on live cells with metasurface-enhanced infrared reflection spectroscopy. *Lab Chip* **21**, 3991–4004 (2021). 34474459 10.1039/d1lc00580d PMC8511245

[16] Dong, L. et al. Nanogapped Au Antennas for Ultrasensitive Surface-Enhanced Infrared Absorption Spectroscopy. *Nano Lett.* **17**, 5768–5774 (2017). 28787169 10.1021/acs.nanolett.7b02736

[17] Caldarola, M. et al. Non-plasmonic nanoantennas for surface enhanced spectroscopies with ultra-low heat conversion. *Nat. Commun.* **6**, 7915 (2015). 26238815 10.1038/ncomms8915 PMC4532885

[18] Yang, X. et al. Polarization-independent metasurfaces based on bound states in the continuum with high Q-factor and resonance modulation. *Opt. Express* **33**, 15682–15689 (2025). 40219475 10.1364/OE.547467

[19] Yang, X., Antonov, A., Hu, H. & Tittl, A. Permittivity-asymmetric qBIC metasurfaces for refractive index sensing. *Nanophotonics* **14**, 5311–5321 (2025). 41426088 10.1515/nanoph-2025-0415 PMC12717935

[20] Aigner, A. et al. Optical control of resonances in temporally symmetry-broken metasurfaces. *Nature* **644**, 896–902 (2025). 40770105 10.1038/s41586-025-09363-7 PMC12390841

[21] Qiu, G. et al. Dual-functional plasmonic photothermal biosensors for highly accurate severe acute respiratory syndrome coronavirus 2 detection. *ACS Nano* **14**, 5268–5277 (2020). 32281785 10.1021/acsnano.0c02439

[22] Toropov, N. A., Houghton, M. C., Yu, D. & Vollmer, F. Thermo-optoplasmonic single-molecule sensing on optical microcavities. *ACS Nano* **18**, 17534–17546 (2024). 38924515 10.1021/acsnano.4c00877 PMC11238588

[23] Houghton, M. C., Kashanian, S. V., Derrien, T. L., Masuda, K. & Vollmer, F. Whispering-gallery mode optoplasmonic microcavities: from advanced single-molecule sensors and microlasers to applications in synthetic biology. *ACS Photonics* **11**, 892–903 (2024). 38523742 10.1021/acsphotonics.3c01570 PMC10958601

[24] Etezadi, D., Warner, J. B. I. V., Lashuel, H. A. & Altug, H. Real-time in situ secondary structure analysis of protein monolayer with mid-infrared plasmonic nanoantennas. *ACS Sens.* **3**, 1109–1117 (2018). 29845861 10.1021/acssensors.8b00115 PMC6133232

[25] Neubrech, F. et al. Resonant plasmonic and vibrational coupling in a tailored nanoantenna for infrared detection. *Phys. Rev. Lett.* **101**, 157403 (2008). 18999639 10.1103/PhysRevLett.101.157403

[26] Wu, C. et al. Fano-resonant asymmetric metamaterials for ultrasensitive spectroscopy and identification of molecular monolayers. *Nat. Mater.* **11**, 69–75 (2012). 10.1038/nmat3161 22081082

[27] Aigner, A., Weber, T., Wester, A., Maier, S. A. & Tittl, A. Continuous spectral and coupling-strength encoding with dual-gradient metasurfaces. *Nat. Nanotechnol.* **19**, 1804–1812 (2024). 39187580 10.1038/s41565-024-01767-2 PMC11638065

[28] Masoudian Saadabad, R., Huang, L. & Miroshnichenko, A. E. Polarization-independent perfect absorber enabled by quasibound states in the continuum. *Phys. Rev. B* **104**, 235405 (2021).

[29] Zhong, H. et al. Efficient polarization-insensitive quasi-BIC modulation by VO_2_ thin films. *Opt Express* **32**, 5862–5873 (2024). 38439302 10.1364/OE.515896

[30] Vaity, P. et al. Polarization-independent quasibound states in the continuum. *Adv. Photonics Res.* **3**, 2100144 (2021).

[31] Baù, E. et al. Spatially Encoded Polaritonic Ultra-Strong Coupling in Gradient Metasurfaces with Epsilon-Near-Zero Modes. *Adv. Mater.* **38**, e10402 (2025). 40936408 10.1002/adma.202510402 PMC12759211

[32] Wang, X., Li, S. & Zhou, C. Polarization-independent toroidal dipole resonances driven by symmetry-protected BIC in ultraviolet region. *Opt Express* **28**, 11983–11989 (2020). 32403699 10.1364/OE.389469

[33] Gao, J. Y. et al. Anisotropic medium sensing controlled by bound states in the continuum in polarization-independent metasurfaces. *Opt Express* **31**, 44703–44719 (2023). 38178534 10.1364/OE.509673

[34] Xiao, S., Qin, M., Duan, J. & Liu, T. Robust enhancement of high-harmonic generation from all-dielectric metasurfaces enabled by polarization-insensitive bound states in the continuum. *Opt Express* **30**, 32590–32599 (2022). 36242316 10.1364/OE.468925

[35] Gölz, T. et al. Revealing mode formation in quasi-bound states in the continuum metasurfaces via near-field optical microscopy. *Adv. Mater.* **36**, 2405978 (2024). 10.1002/adma.202405978 39092689

[36] Paggi, L. et al. Over-coupled resonator for broadband surface enhanced infrared absorption (SEIRA). *Nat. Commun.* **14**, 4814 (2023). 37558692 10.1038/s41467-023-40511-7 PMC10412556

[37] Adato, R., Artar, A., Erramilli, S. & Altug, H. Engineered absorption enhancement and induced transparency in coupled molecular and plasmonic resonator systems. *Nano Lett.* **13**, 2584–2591 (2013). 23647070 10.1021/nl400689q

[38] Aigner, A. et al. Plasmonic bound states in the continuum to tailor light-matter coupling. *Sci Adv* **8**, 4816 (2022). 10.1126/sciadv.add4816 PMC9733921 36490330

[39] Chen, K., Adato, R. & Altug, H. Dual-band perfect absorber for multispectral plasmon-enhanced infrared spectroscopy. *ACS Nano* **6**, 7998–8006 (2012). 22920565 10.1021/nn3026468

