# Supplementary material for: Polarization-independent dielectric gradient near-perfect absorbers for aqueous mid-infrared molecular sensing
Source: Npj Nanophoton. 2026 Apr 13;3(1):25. doi: 10.1038/s44310-026-00121-9 (PMC13076194; doi:10.1038/s44310-026-00121-9)
Supplement: Supplementary file 1 — Supplementary information [file 44310_2026_121_MOESM1_ESM.pdf]

Supporting Information:

Polarization-independent dielectric gradient near-perfect absorbers

for aqueous mid-infrared molecular sensing

*Xingye Yang[1], Tao Jiang[1], Lina Rohrer[1], Jonas Biechteler[1], Andreas Tittl[1]**

1. Nano-Institute Munich, Faculty of Physics, Ludwig-Maximilians-Universität München, Königinstr. 10, 80539 München, Germany.

**E-mail: Andreas.Tittl@physik.uni-muenchen.de*

Contents

Time-resolved stability of a surface adsorbed residual thin water film 2
Estimation of the residual thin water film thickness 3
Illustration of the optical system 4
Coupling regimes on the metasurface 5
Coupling regimes for sensing measurement 7
Influence of incidence angle on the qBIC resonance 9
Circular polarization response of the metasurface 10
Literature comparison for mid-infrared metasurface molecular sensing 11
References 12

Time-resolved stability of a surface adsorbed residual thin water film

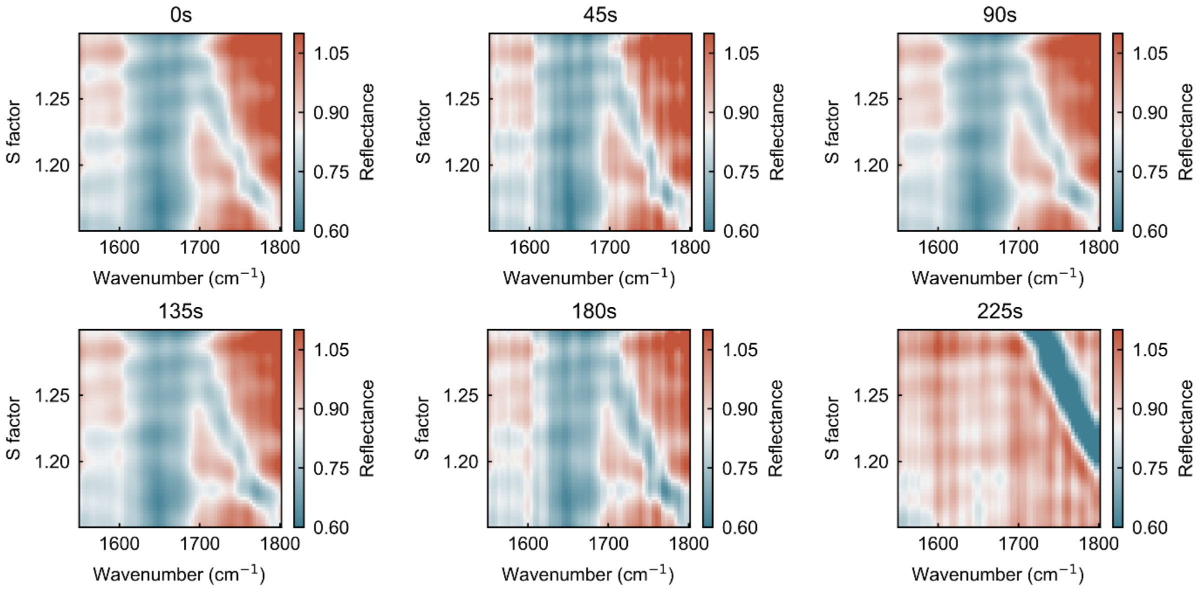

Figure S1. Time-resolved optical spectra recorded at different time delays following the formation of a thin water film adsorbed on the metasurface. Spectra were acquired at 0, 45, 90, 135, 180, and 225 s under identical experimental conditions. The spectral response shows minimal variation within the first 180 s, indicating that the surface-bound water layer persists over this time interval. At 225 s, the spectral features associated with the water film are no longer observed. These measurements indicate a practical temporal window for optical characterization in the presence of a surface-adsorbed water film.

To evaluate the temporal persistence of the thin water film remaining on the metasurface surface, time-resolved optical measurements were performed after the formation of the surface bound residual water layer. Following the establishment of the water film, optical spectra were repeatedly recorded at defined time intervals of 0, 45, 90, 135, 180, and 225 s under identical experimental conditions. The recorded spectra exhibit minimal variation during the initial measurement period up to 180 s, indicating that the residual water layer remains present on the surface over this time interval. Beyond 225 s, the spectral response associated with the water film is no longer observed. These observations suggest that the surface bound residual water layer persists over a finite time window before complete evaporation. It should be noted that the persistence of a surface-bound water film depends on the surface properties of the underlying material. In this work, the surface properties are defined by the CaF_2 spacer layer employed in the metasurface structure. The existence of a stable surface water film over a time window of approximately 180 s provides sufficient duration for reliable optical measurements under aqueous background conditions. This temporal window enables repeated spectral acquisition and analysis before the complete evaporation of the water layer. However, for applications requiring precise quantitative control of bulk water properties (e.g., pH-dependent measurements or concentration-sensitive aqueous analysis), our current configuration is not suitable. Further environmental stabilization would be necessary. This is because even small evaporation-induced changes can alter the effective concentration of the aqueous environment and thereby affect the sensing readout. For such scenarios, the lifetime of the water film could in the future potentially be extended by integrating the metasurface with microfluidic

approaches, e.g. by purging the cell chamber with humidified nitrogen, thereby reducing the evaporation rate. These strategies may allow for improved control over the persistence of the residual water layer.

Estimation of the residual thin water film thickness

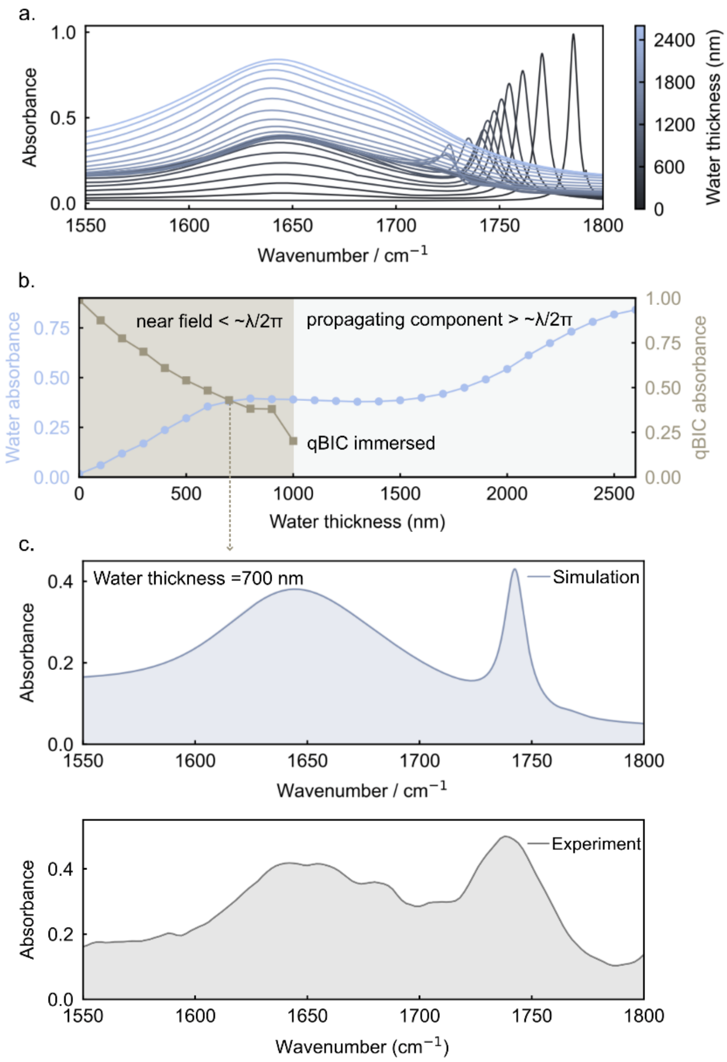

Figure S2. Estimation of the residual thin water film thickness on the metasurface. (a) Simulated absorbance spectra for water thicknesses ranging from 0 to 2400 nm. With increasing water thickness, the water absorption feature becomes stronger, while the qBIC resonance peak is progressively suppressed. (b) Extracted peak values of the water absorption feature (blue) and the qBIC resonance (brown) as a function of water thickness. The water-absorption peak increases with thickness, while the qBIC peak decreases and becomes strongly suppressed at larger thickness. A change in slope of the water-absorption trend is observed around 1000 nm. (c) Comparison between the experimental spectrum and selected simulated spectra, indicating a residual water film thickness of ~700 nm in the experiment.

To estimate the residual thin water-film thickness remaining on the metasurface during the experiment, we performed numerical simulations in which the water thickness was varied from 0 to 2400 nm (Figure S2a). The simulated absorbance spectra show that increasing water thickness strengthens the water absorption feature, while the qBIC resonance peak is progressively reduced, indicating increasing absorption loss introduced by the water layer. For a quantitative comparison, we extracted two peak amplitudes from each simulated spectrum: (i) the peak amplitude of the water absorption band and (ii) the peak amplitude of the qBIC resonance. The extracted peak amplitudes are plotted as a function of water thickness in Figure S2b. The water-absorption peak amplitude (blue curve) increases in an approximately two-regime manner, with a pronounced rise for water thickness below ~1000 nm, followed by a transition region and a continued increase at larger thicknesses. Given that the relevant illumination wavelength is ~6 µm, this behavior suggests a crossover from a thin-film regime where near-field interaction is dominant to a regime where the propagating components contribution becomes important as the water thickness grows. On the other hand, the qBIC peak amplitude (brown curve) decreases with increasing water thickness, and the resonance becomes effectively masked by strong water absorption when the water thickness exceeds 1000 nm. Finally, we compared the simulated spectra, together with the extracted peaks of the water-absorption and the qBIC resonance, to the experimentally measured spectrum (Figure S2c). The agreement is obtained for a water thickness of ~700 nm, for which both the water-absorption peak and the remaining qBIC peak best reproduce the experimental observation. Based on this simulation-experiment matching, the residual water-film thickness on the metasurface in the experiment is estimated to be approximately 700 nm.

Illustration of the optical system

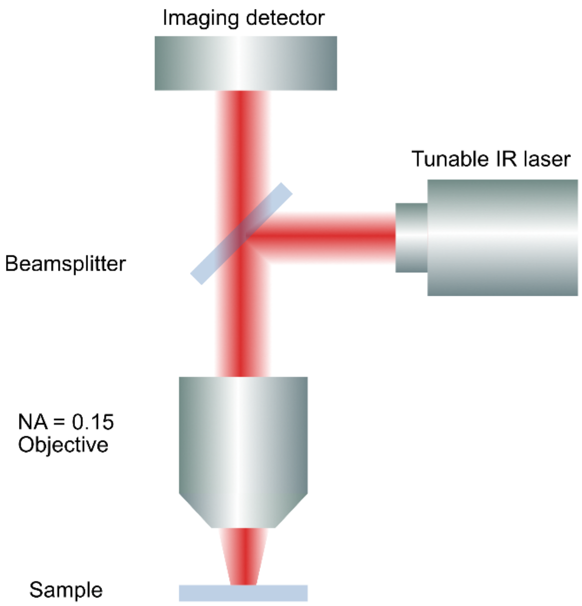

Figure S3. Optical path. Simplified schematic of the measurement setup. Tunable quantum cascade lasers are directed onto the sample via a dichroic mirror and a 4× objective (NA = 0.15). The reflected signal is collected by

the same objective and detected with a 480 × 480 pixel array. The lasers are tuned in 4 cm^{-1} steps across the selected spectral range, and an image is recorded at each wavelength to construct a hyperspectral dataset.

Coupling regimes on the metasurface

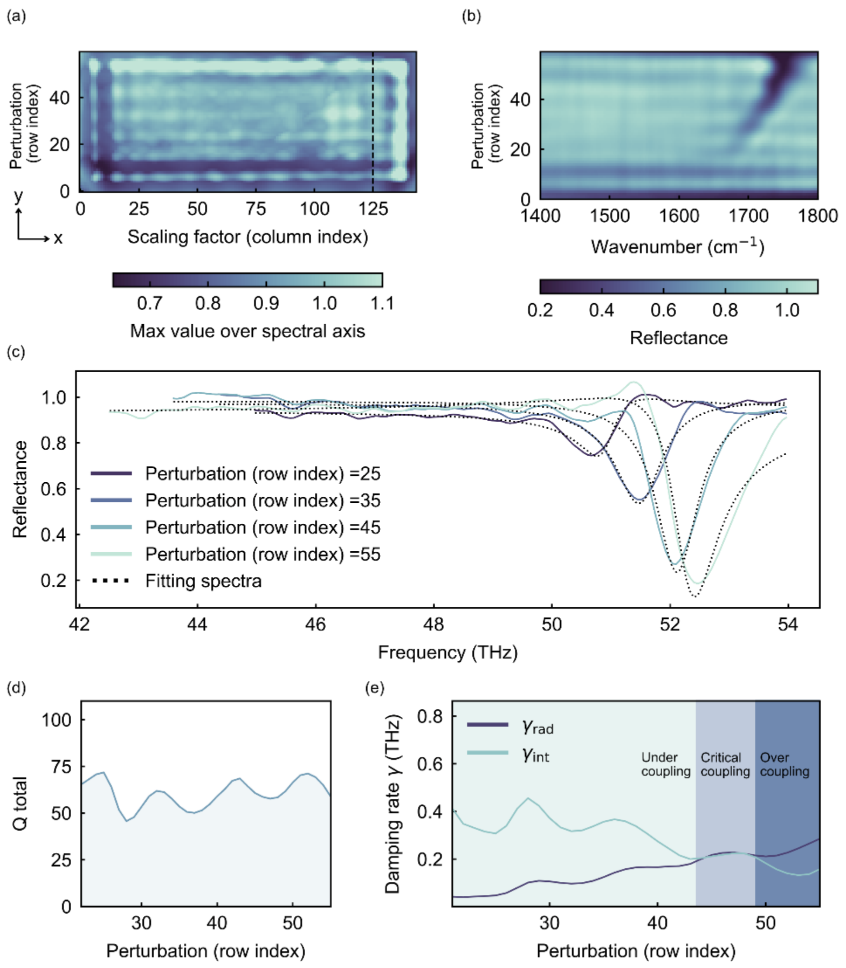

Figure S4. Coupling regimes in the gradient metasurface. (a) Hyperspectal image of the metasurface mapped onto detector pixel coordinates. The metasurface spans approximately 144 pixels along the x direction (scaling factor S) and 60 pixels along the y direction (perturbation factor dx × S). Along the column index, S increases monotonically; along the row index, the perturbation factor increases. The black dashed line marks column index 125 used for spectral extraction. (b) Extracted spectra along column index 125, showing systematic resonance broadening with increasing row index due to increasing perturbation, thus radiative loss. (c) Representative temporal coupled-mode theory (TCMT) fits to the measured spectra. (d) Extracted total Q factor as a function of row index. (e) Extracted radiative loss γ_{rad} and intrinsic loss γ_{int}. The metasurface spans three coupling regimes: under-coupling ($\gamma_{rad} < \gamma_{int}$, rows 0–44), critical coupling ($\gamma_{rad} \approx \gamma_{int}$, rows 44–49), and over-coupling ($\gamma_{rad} > \gamma_{int}$, rows 49–59).

To further clarify the coupling regimes supported by the gradient metasurface, we analyze its spatially resolved spectral response along y direction, as summarized in Figure S4. The fabricated metasurface has a lateral dimension of 600 µm along the x direction and 250 µm along the y direction. The x direction

corresponds to the scaling factor S (monotonically increasing along the column index), while the y direction corresponds to the in-plane perturbation factor (dx × S), which increases along the row index.

The hyperspectral imaging system provides a field of view (FOV) of 2 × 2 mm² with a detector array of 480 × 480 pixels. By converting the physical metasurface size into pixel units, the metasurface spans approximately 144 pixels along x and 60 pixels along y, as illustrated in Figure S4a. Along the column index, the resonance wavelength gradually shifts due to the increasing scaling factor S. Along the row index, the perturbation strength increases monotonically.

To investigate the coupling along the perturbation direction, we fix the column index at 125 (black dashed line in Figure S4a) and extract all spectra along this column. The resulting spectra are shown in Figure S4b. A clear broadening of the resonance linewidth is observed as the row index increases. Within the qBIC framework, this behavior originates from the monotonic increase of radiative loss along the perturbation direction.

All extracted spectra are fitted using temporal coupled-mode theory (TCMT). Representative fitting results are shown in Figure S4c. From these fits, we extract the total quality factor Q_total (Figure S4d) as well as the radiative loss γ_{rad} and intrinsic loss γ_{int} (Figure S4e).

Experimentally, the measured Q values fluctuate around ~70, reaching a maximum of approximately 75. As the row index increases, the perturbation strength increases accordingly, leading to a systematic enhancement of γ_{rad} from 0.07 THz to 0.29 THz. This result highlights the convenience and effectiveness of qBIC engineering in tuning radiative loss.

Figure S4e reveals that the gradient metasurface spans three distinct coupling regimes:

- Under coupling regimes (rows 0–44), where $\gamma_{rad} < \gamma_{int}$
- Critical coupling regimes (rows 44–49), where $\gamma_{rad} \approx \gamma_{int}$
- Over coupling regimes (rows 49–59), where $\gamma_{rad} > \gamma_{int}$

Therefore, a single gradient metasurface simultaneously incorporates under-coupled, critically coupled, and over-coupled regimes within a compact footprint. This spatial encoding of coupling regimes represents a key advantage of the gradient design, enabling systematic exploration of light–matter interaction conditions on a single device.

Coupling regimes for sensing measurement

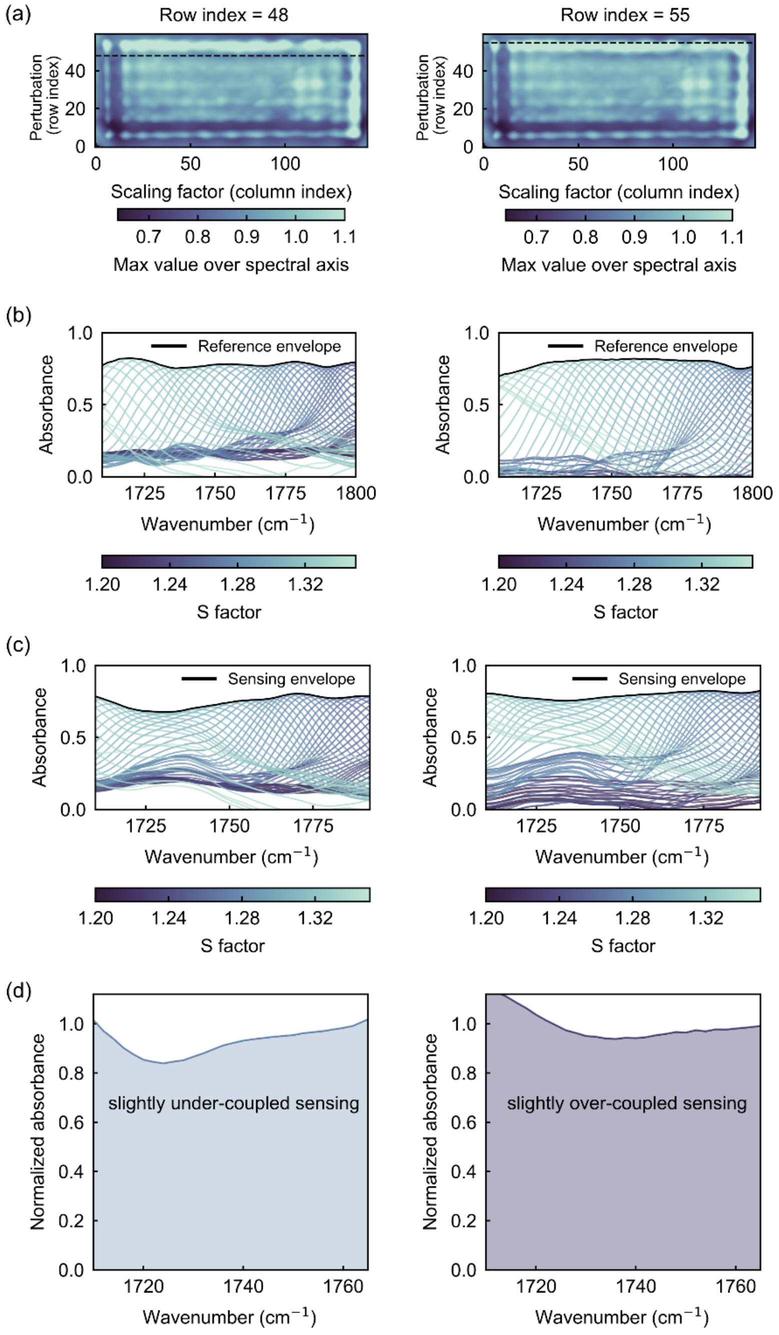

Figure S5. Sensing performance in different coupling regimes. (a) Spatially selected spectra corresponding to two representative coupling regimes identified in Figure S4e. Based on the extracted γ_{rad} and γ_{int}, we select two row indices: row 48 ($\gamma_{rad} \approx \gamma_{int}$, slightly under-coupled) and row 55 ($\gamma_{rad} > \gamma_{int}$, over-coupled). (b) Spectral envelopes of the two selected positions without PMMA coating. (c) Spectral envelopes after deposition of a thin PMMA layer. (d) Normalized envelopes by normalizing (c) and (b). From left to right: slightly under-coupled sensing and slightly over-coupled sensing.

We performed sensing measurements at representative positions from the critical- and over-coupling regimes to compare their sensing performance. The deeply under-coupled region was not included in the quantitative comparison due to its relatively weak resonance strength.

Guided by the extracted loss rates in Figure S4e, we select 2 representative row indices for PMMA sensing. Row index = 48 is close to the critical coupling condition in the uncoated state. After introducing a thin PMMA layer, additional molecular absorption increases the intrinsic loss γ_{int}*, shifting this position into a slightly under-coupled regime. Row index = 55 is located in the over-coupled region.*

In Figure S5a, from left to right panel, the row index increases, which corresponds to a larger perturbation strength and thus a larger radiative loss γ_{rad}*. Figure S5b shows the corresponding spectral envelopes without PMMA, while Figure S5c shows the envelopes after PMMA deposition. By normalizing the envelopes before and after PMMA loading, we obtain the sensing response shown in Figure S5d. From left to right panel, the two cases correspond to slightly under-coupled sensing (row 48) and slightly over-coupled sensing (row 55).*

The two regimes exhibit different sensing characteristics. The slightly under-coupled case provides the better sensing performance, showing a cleaner spectral response and a relatively larger modulation depth. In contrast, the slightly over-coupled regime shows a reduced modulation depth.

Influence of incidence angle on the qBIC resonance

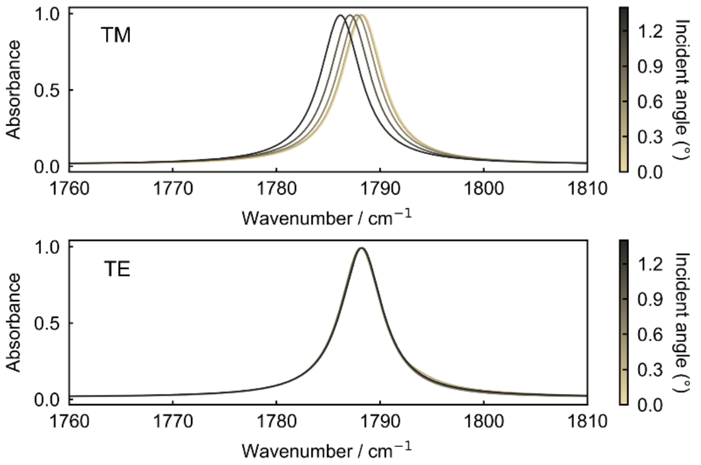

Figure S6. Simulated absorbance spectra under oblique incidence with angles ranging from 0° to 1.4°. (a) TM polarization. A maximum resonance shift of approximately 2 cm^{-1} is observed at the largest incidence angle. (b) TE polarization. The resonance position remains essentially unchanged within the simulated angular range.

To evaluate the influence of incidence angle in the experimental setup, we performed numerical simulations for both TM and TE polarizations with incidence angles varying from 0° to 1.4°, corresponding to the maximum angular spread (±1.33°) for a metasurface spanning 600 µm laterally[1].

The results are shown in Figure S6. For TE polarization (Figure S6b), the resonance position remains essentially unchanged within this angular range, indicating negligible angular sensitivity. For TM polarization (Figure S6a), a gradual resonance shift is observed with increasing angle, reaching approximately 2 cm^{-1} at 1.4° incidence. However, this angular-induced shift is smaller than the experimental spectral sampling interval (4 cm^{-1}) and is negligible compared to the overall resonance tuning induced by the S-factor in the dual-gradient metasurface. Therefore, the finite angular spread in the measurement setup does not significantly affect the interpretation of the experimental results.

Although the angle-induced resonance shift is small, the finite angular distribution still implies that the measured spectrum represents an average over slightly different incidence angles (k-space components). Such angular averaging can broaden the experimentally observed resonance and prevent the reflectance from reaching an ideal zero at a single spectral point. Together with other practical non-idealities (e.g., spatial averaging over the finite pixel area and residual background contributions), this effect can limit the depth of the reflectance dip and thus reduce the experimentally accessible peak absorption. Consistently, while TCMT fitting confirms that the spectra are taken within the critical-coupling window (Fig. S4), the maximum measured absorption saturates at approximately ~0.8 under our experimental conditions (Fig.2).

Circular polarization response of the metasurface

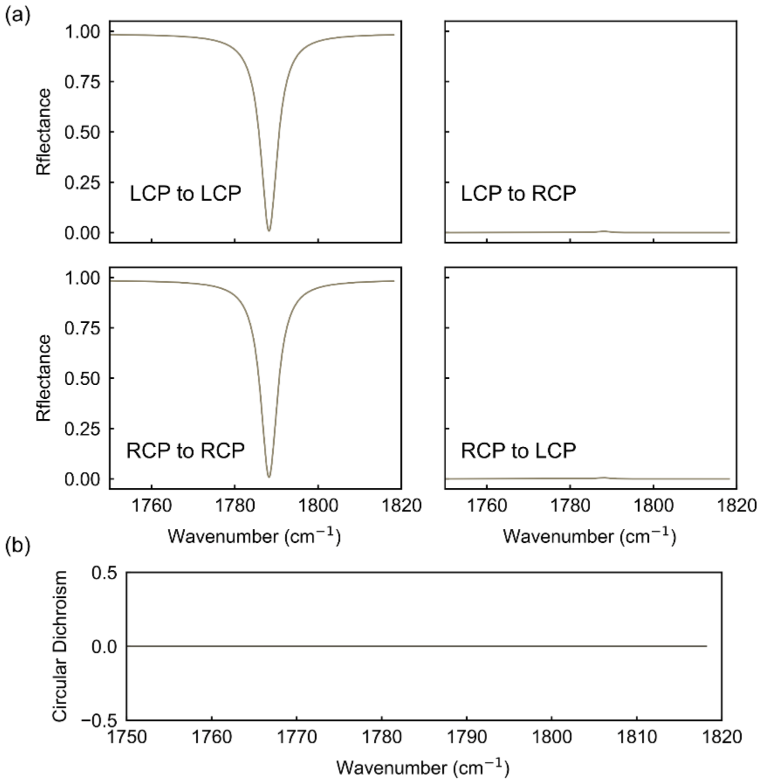

Figure S7. Circular polarization response of the metasurface. (a) Simulated reflectance spectra under circularly polarized excitation, including co-polarized (LCP→LCP, RCP→RCP) and cross-polarized (LCP→RCP, RCP→LCP) channels. (b) Calculated circular dichroism (CD), defined as $CD(\omega)=(R_{RR}(\omega)-R_{LL}(\omega))/(R_{RR}(\omega)+R_{LL}(\omega))$. The CD remains near zero across the resonance, confirming polarization-independent behavior under circularly polarized illumination.

To further verify the polarization-independent response under circularly polarized illumination, we performed numerical simulations using circular polarization. Specifically, we calculated the reflectance for four polarization channels: LCP (Left-Circularly Polarized)-to-LCP, LCP-to-RCP (Right-Circularly Polarized), RCP-to-RCP, and RCP-to-LCP, as shown in Figure S7a.

The results demonstrate that the co-polarized channels (LCP→LCP and RCP→RCP) exhibit identical resonance responses, while the cross-polarized channels (LCP→RCP and RCP→LCP) remain negligible across the spectral range. We calculated the circular dichroism (CD), defined as $CD(\omega)=(R_{RR}(\omega)-R_{LL}(\omega))/(R_{RR}(\omega)+R_{LL}(\omega))$, where $R_{LL}(\omega)$ and $R_{RR}(\omega)$ denote the co-polarized reflectance under left- and right-circularly polarized incidence, respectively. The calculated CD spectrum is shown in Figure S7b. As observed, the CD value remains essentially zero across the resonance, confirming that the metasurface exhibits negligible circular dichroism. This result is consistent with the C_4 symmetry of the unit cell, which ensures polarization-independent excitation. Physically, circular polarization can be decomposed into two orthogonal linear polarization components (x and y directions) with equal amplitude and a ±90° phase difference. Since the metasurface response is identical for orthogonal linear polarizations, the response under LCP and RCP illumination is also identical.

Literature comparison for mid-infrared metasurface molecular sensing

Table S1. Representative literature comparison for mid-infrared metasurface molecular sensing.
Summary of sensing platform, resonance quality factor, analyte type, sensing environment, and reported sensing metrics for representative studies.

**Platforms**	**Quality factor (in experiment)**	**Sensing background**	**Analytes**	**Sensing metrics**
Our work: Dielectric gradient absorber metasurface	~75	Air	~4 nm thick PMMA	Envelope modulation: 20%
		Thin H_2O film	~7 nm thick PMMA	Envelope modulation: 30%
Dielectric metsurface[2]	~80	D_2O	AzoPC	Envelope modulation: ~15%
Metallic dual-band perfect absorber[3]	~15	Air	~4 nm thick PMMA	Envelope modulation: ~30%
Dielectric gradient metasurface[1]	~110	Air	1.5 nm to 200 nm thick PMMA	$A = -\log(R/R_0)$ Max(A): ~0.3 to 1
Metallic gradient metasurface[4]	~30	Air	Several-nanometer thin PMMA	Max(A): 0.5
		Bulk H_2O		Max(A): 0.1
Dielectric gradient metasurface[4]	~70	Air		Max(A): 0.6
		Bulk H_2O		Max(A): 0
Dual resonances metallic metasurface[5]	Less than 10	Bulk H_2O	Dynamic lipid membrane	Max(A): 0.01
Metallic absorber metasurface[6]	~45	Air	~1.5 nm thick PMMA	Absorbance difference: 0.05

References

1. Aigner, A., Weber, T., Wester, A., Maier, S. A. & Tittl, A. Continuous spectral and coupling-strength encoding with dual-gradient metasurfaces. *Nat. Nanotechnol. 19, 1804–1812 (2024).*

2. Barkey, M. *et al.* Pixelated High-Q Metasurfaces for in Situ Biospectroscopy and Artificial Intelligence-Enabled Classification of Lipid Membrane Photoswitching Dynamics. *ACS Nano* 18, 11644–11654 (2024).

3. Chen, K., Adato, R. & Altug, H. Dual-Band Perfect Absorber for Multispectral Plasmon-Enhanced Infrared Spectroscopy. *ACS Nano* 6, 7998–8006 (2012).

4. Jiang, T. *et al.* A Comparative Analysis of Plasmonic and Dielectric Metasurface Sensing Platforms Powered by Bound States in the Continuum. *Adv. Funct. Mater.* 36, e16021 (2025).

5. Rodrigo, D. *et al.* Resolving molecule-specific information in dynamic lipid membrane processes with multi-resonant infrared metasurfaces. *Nat. Commun.* 9, 2160 (2018).

6. Wang, J., Weber, T., Aigner, A., Maier, S. A. & Tittl, A. Mirror-Coupled Plasmonic Bound States in the Continuum for Tunable Perfect Absorption. *Laser Photon. Rev.* 17, 2300294 (2023).
